# The potential oncogenic and MLN4924-resistant effects of CSN5 on cervical cancer cells

**DOI:** 10.1186/s12935-021-02078-5

**Published:** 2021-07-12

**Authors:** Huilin Zhang, Ping He, Qing Zhou, Yan Lu, Bingjian Lu

**Affiliations:** 1 Department of Surgical Pathology, Women’s Hospital, School of Medicine, Zhejiang University, Hangzhou, Zhejiang China; 2 Shanghai Jiao Tong University School of Medicine, Shanghai, 200025 China; 3 Center for Uterine Cancer Diagnosis & Therapy Research of Zhejiang Province, Women’s Reproductive Health Key Laboratory of Zhejiang Province, and Department of Gynecologic Oncology, Women’s Hospital, Zhejiang University School of Medicine, Hangzhou, 310029 Zhejiang China; 4 Institute of Translational Medicine, Zhejiang University School of Medicine, Hangzhou, China

**Keywords:** Cervical cancer, CSN5, MLN924, Neddylation

## Abstract

**Background:**

CSN5, a member of Cop9 signalosome, is essential for protein neddylation. It has been supposed to serve as an oncogene in some cancers. However, the role of CSN5 has not been investigated in cervical cancer yet.

**Methods:**

Data from TCGA cohorts and GEO dataset was analyzed to examine the expression profile of CSN5 and clinical relevance in cervical cancers. The role of CSN5 on cervical cancer cell proliferation was investigated in cervical cancer cell lines, Siha and Hela, through CSN5 knockdown via CRISPR–CAS9. Western blot was used to detect the effect of CSN5 knockdown and overexpression. The biological behaviors were analyzed by CCK8, clone formation assay, 3-D spheroid generation assay and cell cycle assay. Besides, the role CSN5 knockdown in vivo was evaluated by xenograft tumor model. MLN4924 was given in Siha and Hela with CSN5 overexpression.

**Results:**

We found that downregulation of CSN5 in Siha and Hela cells inhibited cell proliferation in vitro and in vivo, and the inhibitory effects were largely rescued by CSN5 overexpression. Moreover, deletion of CSN5 caused cell cycle arrest rather than inducing apoptosis. Importantly, CSN5 overexpression confers resistance to the anti-cancer effects of MLN4924 (pevonedistat) in cervical cancer cells.

**Conclusions:**

Our findings demonstrated that CSN5 functions as an oncogene in cervical cancers and may serve as a potential indicator for predicting the effects of MLN4924 treatment in the future.

## Background

The incidence and the mortality of cervical carcinoma have dropped markedly in developed countries owing to the wide adoption of populational screening program and recent human papillomavirus (HPV) vaccination. However, cervical carcinoma remains the fourth most common cancer worldwide in women with an estimated 570,000 new cases in 2018 [1–3]. In China, cervical carcinoma had an estimated incidence of 9.89 and mortality of 3.05 per 100,000 in 2015, ranking the leading cancers in the female genital tract [4]. The long-term survival rate remains low in women with advanced stage and cancer metastasis [5, 6]. Approximately 20% of patients may recur despite the combined surgery, chemotherapy and radiotherapy [7]. At present, the combination of platinum, paclitaxel and bevacizumab is suggested to be the first choice of metastatic cervical cancer [8–10], but the prognosis remains poor in general [11–14]. It is a pressing need to discover potential therapeutic and diagnostic biomarkers to improve survival in women with cervical cancers.

The activity of Skp1-cullin1-Fbox (SCF) E3 ligases are regulated by neddylation and deneddylation, which further regulates protein stability. Neddylation, a post-translational modification, plays vital roles in various physiological processes by adding ubiquitin-like protein NEDD8 to cullin1. Neddylation blockade is suggested to be an attractive anticancer therapy [15–17]. MLN4924 (pevonedistat) is a small molecular inhibitor that effectively disrupts neddylation by targeting NEDD8-activating enzyme (NAE) specifically. MLN4924 has been reported to have anticancer effects on various cancers, including cervical cancer [17, 18]. Now, it is applied as a promising anti-cancer drug in clinical I/II trials [19]. However, molecules that modulate the pharmacological effects of MLN4924 have not been fully investigated yet.

Deneddylation is the reversed process of neddylation, mainly regulated by Cop9 signalosome (CSN), which is essential for protein turnover [20]. CSN5, also named as c-Jun activation domain-binding protein-1 (Jab1), is the fifth component of the CSN complex. Accumulative evidence has indicated that CSN5 may be a prognostic indicator for a variety of cancers, such as ovarian cancer [21, 22], colorectal cancer [23], pancreatic cancer [24] and breast cancer [25]. However, to date, little is known about the role of CSN5 in cervical cancer. Being the only component with enzyme activity, CSN5 is critical in regulating SCF-mediated protein degradation [20, 26]; therefore, we hypothesize that CSN5 overexpression may influence the therapeutical effects of MLN4924 in cervical cancer.

In this study, we aim to investigate the role of CSN5 in cervical cancer and the potential effects of CSN5 on MLN4924 in cancer treatment. We hope that our findings will contribute to our understanding towards the role of CSN5 and MLN4924 in cervical cancers.

## Material and methods

### Dataset analysis

The data of RNA expression and survival in cervical cancers were retrieved from public datasets: TCGA cohorts (The Cancer Genome Atlas) and GEO dataset (GSE6791 & GSE7410). We compared the difference of overall survival between 44% patients with highest and those with lowest CSN5 expression in TCGA datasets.

### Cell culture and treatment

Siha and Hela cells were grown in MEM (GIBCO, Australia) while human embryonic kidney 293T (HEK293T) in DMEM (GIBCO, Australia), supplemented with 10% FBS (GIBCO, Australia), penicillin (100 U/mL) and streptomycin (100 ng/mL) (GIBCO, Australia) at 37 °C in 5% CO_2_. MLN4924 (MCE, USA) was added to investigate its role in cervical cancer cells.

### Plasmids, shRNAs, CRISPR-CAS9 and transfections

The gRNA sequence of CSN5 was designed via https://zlab.bio/guide-design-resources. It was inserted into LentiCRISPR v2. The CSN5 targeting guide RNAs (gRNA) sequence is CACCGGCCTTGAAAATGCAATCGGG. CSN5 overexpression vector was constructed by inserting correspondent CDS into PLVX-puro vector. The E6/E7 of HPV16 targeting shRNA is GGTTGTGCGTACAAAGCAC and GGACAGAGCCCATTACAAT. The E6/E7 of HPV18 targeting shRNA is GCATGGACCTAAGGCAACA and GCGCTTTGAGGATCCAACA. The virus for shRNAs, gRNAs, and CSN5 were packaged with PSAX2 and PMDG through lipo3000 (Invertrogen, America) in 293T cells. After cells were infected by the virus for 48 h, puromycin was applied to select positive cells.

### Real-time quantitative PCR

Total RNA was extracted using Trizol according to manufacturer’s instructions. 1 μg RNA was digested with gDNA wiper to remove DNA and then was subjected to reverse transcription to synthesize cDNA using HiScript II Reverse Transcriptase (Vazyme, China). Subsequently, the cDNA products were used in SYBR Green PCR Master Mix (Takara, Japan) based Real-time quantitative PCR. The forward sequence of CSN5 is ACCCAAAGGGCTACAAACCTC and the reverse sequence is AGCTCAAGCAATTTGCGATCC. GAPDH was used as reference gene (forward: GTCTCCTCTGACTTCAACAGCG; reverse: ACCACCCTGTTGCTGTAGCCAA). The relative amount of CSN5 was calculated though the 2^−△△Ct^ Method.

### Western blot analysis

Western blot was used to detect the effect of CSN5 knockdown and overexpression. Cell lysates were separated by SDS-polyacrylamide gel, transferred to nitrocellulosemembrane (GE Amersham, Buckinghamshire, UK), blocked by 5% nonfat milk in phosphate-buffered saline and immunoblotted with the indicated antibodies. Different blots were incubated with a panel of antibodies against CSN5 (Proteintech, Cat# 27511-1-AP, 1:2000), cullin1 (Proteintech, Cat# 12895-1-AP, 1:2000), β-actin (Haro Life, Cat# 1030300012 1;1000), PARP (Cell signaling, Cat# 9542, 1;1000) and p27 (Cell signaling, Cat# 3686,1:1000). Followed by incubation in horseradish peroxidase (HRP)-linked secondary antibodies (Cell signaling, Cat# 7074, 1:2000) at room temperature for 1 h. Blots were then visualized by Immobilon Western Chemiluminescent HRP substrate kit (Merck Millipore) according to the manufacturer’s instructions.

### Cell cycle analysis

The cells were collected and fixed overnight in 75% cold ethanol at -20 ℃. Then the cells were washed twice with cold phosphate-buffered saline solution and labeled with propidium iodide (PI) /RNase Staining Buffer (BD, Cat# 550825) according to the manufacturer’s instructions. The PI labeled cells were analyzed immediately after staining with a flow cytometer.

### Cell proliferation and colony formation assay

Cell proliferation was evaluated via the rate of cell growth. CCK8 kit (MCE, USA) was applied to test the cell growth rate. Siha and Hela cells were seeded in 96-well plates with 2000 cells per well in 100 μl medium. Subsequently, CCK-8 reagent was added into wells at D0, D1, D2, D3, D4 and D5, respectively. After 1.5 h of incubation, the absorbance was measured at a wavelength of 450 nm. For colony formation assay, 500 cells were planted in the six-well or twelve-well plates. About 10 days later, cell colonies formed and stained with 0.1% crystal violet for 10 min, washed, photographed and counted.

### 3-D Spheroid generation and viability assays

3-D Spheroid formation assay was performed as reported previously [27, 28]. Briefly, the cells (500/well) were seeded in a low-adhesion U-bottom microplate (Costar, Cat# 7007). The spheroids were generated after 24 h and MLN4924 (0.5 μM) were added. Spheroid viability was analyzed utilizing CellCounting-Lite® 3D Luminescent (Vazyme, Cat# DD1102) according to the manufacturer’s instructions.

### Animal experiments

Hela cells (1.5 × 10^6^) with or without CSN5 stable knockdown were inoculated subcutaneously into five-week-old female nude mice. Mice were monitored every 2 days for tumor growth. After 25 days of inoculation, mice were placed in a chamber for euthanasia and 100% carbon dioxide (CO_2_) was then introduced with a fill rate of 30–40% of the chamber volume per minute. Compressed CO_2_ gas in cylinders was used as the source of CO_2_ and it allows the inflow of gas to the induction chamber to be controlled. Mice were sacrificed by treating with 100% CO_2_ for 2–3 min. The harvest tumors were weighted. Animal care and experiments were performed strictly according to the Guide for the Care and Use of Laboratory Animals (NIH publication 80–23, revised 1996), and approved by the Institutional Laboratory Animal Welfare and Ethics Board (Zhejiang University, China).

### Statistical analysis

The SPSS 16.0 (SPSS, Inc., Chicago, IL, USA) software package was applied for the statistical analyses. *Unpaired student’s t-test* and *two-way ANOVA* were applied to show the differences. The statistical threshold was set at 0.05 (two-sided).

## Results

### Elevated CSN5 expression predicts poor prognosis in cervical cancer

**Fig. 1:**
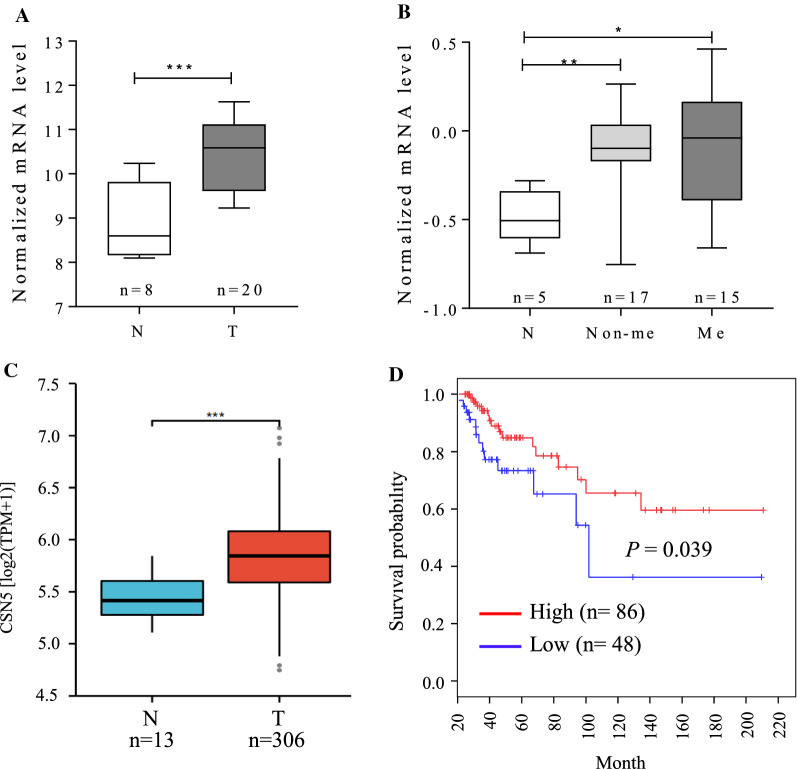
Elevated CSN5 expression in cervical cancer predicts poor prognosis. The data of CSN5 expression in GSE6791 (**A**), GSE7410 (**B**) and TCGA (**C**) cervical cancer patient cohorts. In TCGA dataset, Kaplan–Meier analysis showed that the overall survival was worse in cervical cancer patients with high expressions of CSN5 than those with low expression (**D**)

We analyzed the expression of CSN5 in cervical cancer by searching the public database. From the GSE6791, GSE7410 and TCGA datasets, we found that the level of CSN5 expression was significantly up-regulated in cervical cancers compared with normal tissue (Fig. 1A–C). However, CSN5 expression showed no significant alteration between non-metastasis and metastasis cancers in the GSE7410 dataset (Fig. 1B). The TCGA data indicated that elevated CSN5 expression was associated with unfavorable clinical outcomes in long-term survival cancer patients (Fig. 1D).

### Knockdown of CSN inhibits cervical cancer cell growth in vitro

**Fig. 2:**
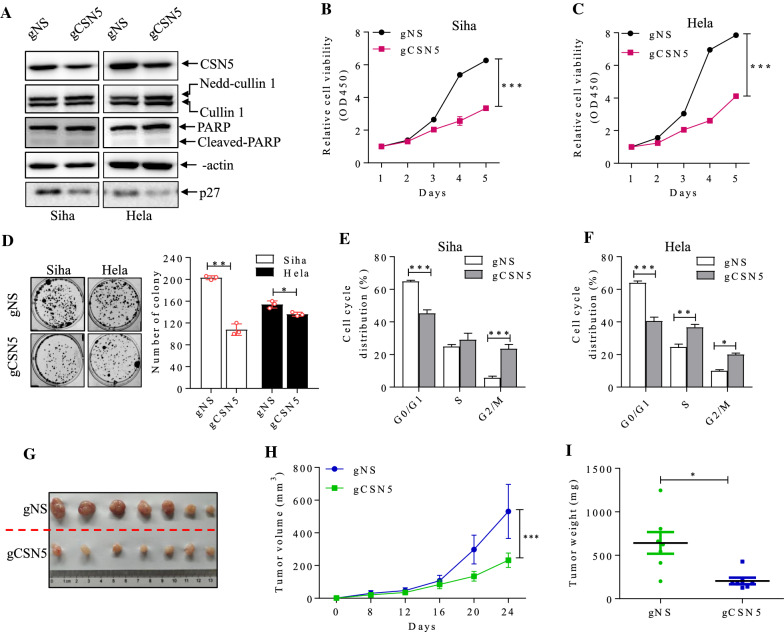
CSN5 knockdown inhibits cell growth in vitro and in vivo. Depicted are Western blot analysis of indicated proteins (**A**), CCK8 tests (**B**, **C**), clone formation (**D**) and cell cycle analysis (**E**, **F**) in Siha and Hela cells with CAS9 CSN5 knockdown (gCSN5), and a non-specific guide RNA as control (gNS). (G, H, I) Hela cells with gCSN5 and controls were subcutaneously injected into nude mice (n = 7 mice for each group). Tumor volumes were measured at different time points and tumors were harvested and weighed after 24 days. (Mean with S.E.M. *, p < 0.05; **, p < 0.01; ***, p < 0.001)

To explore the role of CSN5 in cervical carcinogenesis, lentiviruses encoding CAS9 and gRNAs (gCSN5) was designed to knockdown CSN5 along with a non-specific gRNA (gNS) in cervical cancer cells (Fig. 2A). We found that CSN5 downregulation impaired deneddylation owing to elevated neddylated cullin1 (Fig. 2A). Notably, we observed that CSN5 knockdown inhibited Siha (Fig. 2B) and Hela cell (Fig. 2C) growth. The colony formation assay showed that deletion of CSN5 suppressed clone forming ability in Siha and Hela cells (Fig. 2D). Flow cytometric analyses demonstrated that CSN5 deprivation caused Siha and Hela cell cycle arrest in G2/M phase and S phase in Siha and Hela cells (Fig. 2E, F). However, CSN5 knockdown did not significantly influence the expression of cyclin A2, cyclin B1, cyclin D1, cyclin D3, cyclin E1, and cyclin E3 proteins, the important regulators of cell cycle (data not shown). Intriguingly, CSN5 knockdown alleviated p27 expression while CSN5 overexpression upregulated p27 level (Fig. 2A & 4A). CSN5 has been reported to participate in cancer cell death [29], but we couldn’t determine the alteration of cleaved PARP1 in both Siha and Hela cells in CSN5-depleted cells (Fig. 2A). To further confirm the potential oncogenic role of CSN5 in cervical cancer, we also investigated the role of CSN5 in vivo. We subcutaneously injected the established CSN5 deficient Hela cells into BALB/c-nude mice. The size, growth rate and tumor weight in gCSN5 xenografts decreased significantly, compared to the gNS group (Fig. 2G–I).

### Overexpression of CSN5 in CSN5-deficient cells rescues cell growth

**Fig. 3:**
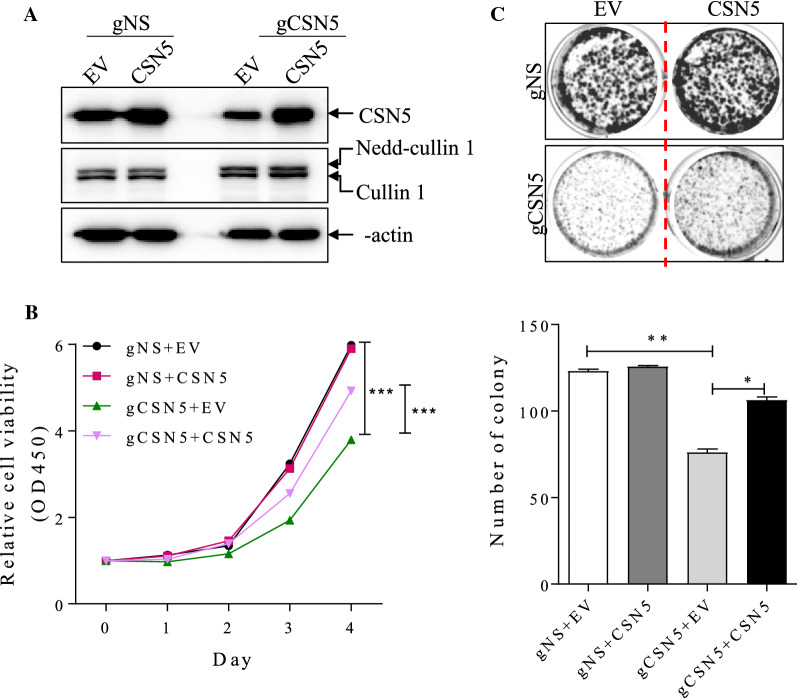
CSN5 overexpression rescues cell growth in CSN5-deficient cells. (**A**) Western blot analysis of indicated proteins in CSN5 deficient Siha cells that were further transduced by CSN5 and empty vector (EV) as a negative control. The rescue effects were shown by (**B**) CCK8 and (**C**) Clone formation analysis. (Mean with S.E.M.. *, p < 0.05; **, p < 0.01; ***, p < 0.001)

We constructed CSN5 overexpression lentivirus vector and then introduced it into Siha cell with or without CSN5 stable knockdown by lentiviruses encoding CSN5 while Siha cell with empty vector (EV) were taken as a negative control. CSN5 re-expression (Fig. 3A) successfully rescued cell growth in Siha cells with CSN5 depletion (Fig. 3B). Consistently, the clone formation assay showed that CSN5 overexpression had more and larger clones than control cells (Fig. 3C).

### Overexpression of CSN5 increases resistance to MLN4924 in cervical cancer cells

**Fig. 4:**
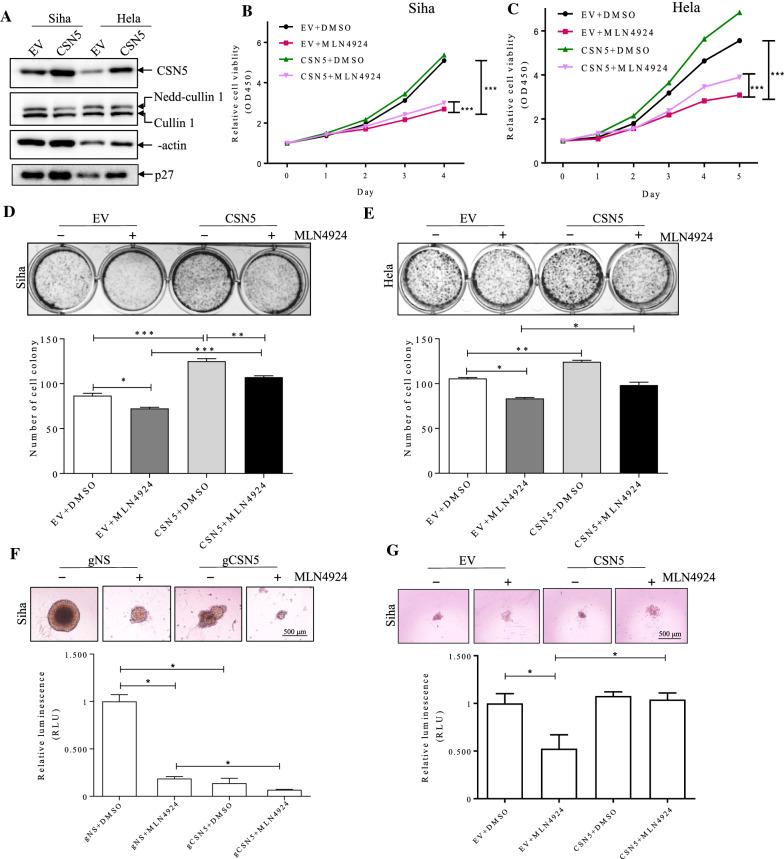
Overexpression of CSN5 increases resistance to MLN4924 in Siha and Hela cells. **A** Western blot analysis of indicated proteins in Siha and Hela cells transduced by CSN5 ad EV as a negative control. These cells were treated with 0.75 μM MLN4924 in CCK8 assay (**B**) or 0.5 μM MLN4924 in clone formation assay (**C**) and spheroid generation assay (**F**, **G**). (Mean with S.E.M. *, *p* < 0.05; **, *p* < 0.01; ***, *p* < 0.001)

MLN4924, a highly selective small-molecule inhibitor of NAE, can impair neddylation. It has been regarded as a promising anti-cancer drug by some preclinical trials. We found that depletion of E6/E7 exerted little change of neddylation in Siha and Hela cells (data not shown), implicating that the anti-activity of MLN4924 in cervical cancers was independent from E6/E7 oncoproteins. We next sought to test whether CSN5 overexpression confers resistance in MLN4924 treatment. CSN5 overexpression was associated with decreased neddylated-cullin1 in Siha and Hela cells (Fig. 4A). MLN4924 treatment caused growth arrest of Siha and Hela cells by showing significant lower proliferation rate (Fig. 4B, C) and fewer, smaller clones (Fig. 4D, E). Importantly, CSN5 overexpression countered the effects MLN4924 treatment in cervical cancer cells (Fig. 4B–E). The 3-D Spheroid formation assay indicated that CSN5 depletion or overexpression could enhance or counteract the anti-cancer effects of MLN4924 treatment in Siha cells, respectively (Fig. 4F, G). However, these biological alterations were not significant in Hela cells (data not shown).

## Discussion

To the best of our knowledge, our work is the first report on the role of CSN5 in cervical cancer in vitro and in vivo. In this study, we found that the level of CSN5 was elevated in different public patient cohorts and higher level of CSN5 predicted dismal prognosis. CSN5 knockdown in cervical cancer cells impaired proliferation both in vitro and in vivo. Additionally, the impaired proliferation was associated with cell cycle arrest rather than apoptosis inhibition. These data suggested that CSN5 might play a tumor-promoting role in cervical cancers as reported previously in other cancers [23, 25].

Compelling data demonstrated that CSN5 facilitated cancer cells survival by involving the process of cell proliferation, apoptosis and DNA-damage response via regulating the stability of certain regulatory proteins including some key tumor suppressors [23, 30–32]. Of note, high level of CSN5 expression was usually correlated with p27 downregulation in various cancers [22, 25, 29, 33]. In serious ovarian cancer, CSN5 interacted with p27 and induced p27 degradation [21]. Moreover, CSN5 could specifically induce p27 translocation from nuclear to cytoplasm and accelerate its degradation [23, 24, 34]. However, we here found the opposite results in cervical cancer cells: CSN5 overexpression upregulated p27 level while depletion downregulated p27 level. It is possible that the elevated p27 by CSN5 overexpression could be a compensatory effect in this context. Additionally, we also found that CSN5 knockdown showed no effects on several important regulators in cervical cancer, including cyclin D1 and cyclin E2. Further studies are needed to investigate the molecular mechanisms of CSN5 underlying proliferation inhibition in cervical cancer cells.

Neddylation inhibitors are promising drugs in cancer treatment, such as MLN4924, which has been found to be effective in cervical cancers [18]. It is well-known that high-risk human papillomavirus (HPV) infection is the cardinal cause of cervical cancers [35]. HPV16 and HPV18 are the main types of high-risk HPV, which can cause cervical cancer including parent patients of Siha and Hela cells by integrating with HPV E6 and E7 oncoproteins [36]. We here found that E6/E7 downregulation exerted little effects on neddylation although MLN4924 are effective in killing Siha and Hela cells in keeping with previous reports [18]. Therefore, we suggest that the effects of MLN4924 on cervical cancers might be independent of E6/E7. More importantly, we showed that CSN5 overexpression exhibited MLN4924 resistance as evidenced by increased cell growth rate and clone formation ability, which are indicators of chemoresistance [37]. The effects of CSN5 overexpression on MLN4924 resistance were further validated by 3-D Spheroid formation assay in Siha cells rather than in Hela cells. Similar findings have been observed with regard to 3-D spheroid formation assay in Hela cells with other anti-cancer drugs [38, 39]. The high matrix metalloproteinnase (MMP) expression may account for the specific alterations in Hela cell spheroid formation [40]. Theoretically, CSN5 overexpression suppressed neddylation by enhancing deneddylation activity while MLN4924 downregulated neddylation by inhibiting NAE [41].

## Conclusion

In conclusion, we suggest that elevated CSN5 expression indicates poor clinical outcome and confers to MLN4924 resistance in cervical cancers. Further work is critically required to consolidate these findings in clinical settings and to explore the underlying molecular mechanisms of CSN5 expression underlying MLN4924 resistance in cervical cancer cells.

## Data Availability

The datasets used and/or analyzed during the current study are available from the corresponding author on reasonable request.

## Abbreviations

HPV: Papillomavirus
SCF: Skp1-cullin1-Fbox
NAE: NEDD8-activating enzyme
CSN: Cop9 signalosome
Jab1: Jun activation domain-binding protein-1
HEK293T: Human embryonic kidney 293T
gRNA: Guide RNAs
HRP: Horseradish peroxidase
PI: Propidium iodide
